# Role of Osteoplastic Frontal Sinus Obliteration in the Era of Endoscopic Sinus Surgery

**DOI:** 10.1155/2012/501896

**Published:** 2012-10-16

**Authors:** Joshua B. Silverman, Stacey T. Gray, Nicolas Y. Busaba

**Affiliations:** ^1^Department of Otolaryngology—Head and Neck Surgery, SUNY Downstate Medical Center, Brooklyn, NY 11203, USA; ^2^Department of Otolaryngology—Head and Neck Surgery, Massachusetts Eye and Ear Infirmary, 243 Charles Street, Boston, MA 02114, USA; ^3^Department of Otology and Laryngology, Harvard Medical School, Boston, MA 02115, USA

## Abstract

*Objective*. Determining the indications for osteoplastic frontal sinus obliteration (OFSO) for the treatment of inflammatory frontal sinus disease. *Study Design*. Retrospective case series from a single tertiary care facility. *Methods*. Thirty-four patients who underwent OFSO for chronic frontal sinusitis (*n* = 23) and frontal sinus mucocele (*n* = 11) comprised our study group. Data reviewed included demographics, history of prior frontal sinus operation(s), imaging, diagnosis, and operative complications. *Results*. The age range was 19 to 76 years. Seventy percent of patients with chronic frontal sinusitis underwent OFSO as a salvage surgery after previous frontal sinus surgery failures, while 30% underwent OFSO as a primary surgery. For those in whom OFSO was a salvage procedure, the failed surgeries were endoscopic approaches to the frontal sinus (69%), Lynch procedure (12%), and OFSO outside this study period (19%). For patients with frontal sinus mucocele, 72% had OFSO as a first-line surgery. Within the total study population, 15% of patients presented for OFSO with history of prior obliteration, with a range of 3 to 30 years between representations. *Conclusions*. Osteoplastic frontal sinus obliteration remains a key surgical treatment for chronic inflammatory frontal sinus disease both as a salvage procedure and first-line surgical therapy.

## 1. Introduction

Chronic frontal rhinosinusitis remains a highly prevalent disease with significant associated morbidity despite advances in pharmacology, physiology, and technology [1]. Current frontal sinus surgery has evolved to include minimally invasive endoscopic techniques in addition to more traditional open frontal sinus obliteration [2]. However, the proper utilization of each of these different techniques remains a controversial topic. The osteoplastic flap frontal sinus obliteration (OFSO) procedure popularized by Montgomery in the 1960s has historically been accepted as the gold standard for treatment of frontal sinus disease [2]. The main impetus for developing this technique was the high failure rate associated with earlier external techniques such as the Lynch and Lothrop procedures, which produced short-term patency rates up to 90% but failed at least an additional 20% over a seven-year follow-up period [2]. This led to the prevailing dogma in the otolaryngology specialty that trauma to the mucosa of the frontal recess inevitably leads to scarring and obstruction of the frontal sinus outflow tract. Hence, any surgical manipulation of the frontal recess was discouraged. Even though OFSO with fat obliteration remains a definitive treatment, this procedure also has a reported long-term failure rate of up to 18% [3]. In addition, there can be significant associated morbidity including frontal bossing, supraorbital neuralgia, and donor site complications after abdominal fat grafting. Difficulties interpreting post-operative imaging can also complicate management of patients with persistent symptoms after frontal sinus obliteration. Due to these potential complications in addition to the continued advancement in endoscopic techniques, different procedures to widen the frontal sinus outflow tract while minimizing scarring have been developed. Endoscopic approaches have been created to simulate the external technique pioneered by Lothrop in the 1800s in which there was resection of the medial frontal sinus floor, superior nasal septum, and intersinus septum [1]. In 2001, Weber et al. described a series of three endoscopic techniques for widening the frontal sinus ostium [4]. The Draf Type III technique has also been termed frontal sinus drillout or endoscopic modified lothrop procedure (EMLP), as described by Gross et al. in 1995 [5].

 Management of frontal sinus disease may be the most challenging aspect of paranasal sinus disease management, and the proper choice of surgical technique continues to be a highly prolific topic in the otolaryngology literature. Despite the evolution of advanced endoscopic techniques for frontal sinus surgery, the open approach of OFSO continues to be a reasonable option given its long-term proven success. In general, currently accepted indications for OFSO include chronic frontal sinusitis after failed endonasal surgery, frontal sinus muco(pyo)cele, severe frontal bone fractures, especially involving the frontal duct area and tumors [6]. The objective of this paper is to determine whether there is still a role for OFSO in the era of advanced endoscopic sinus surgery in the management of inflammatory frontal sinus disease by analyzing our own experience with OFSO over a 12-year period and comparing our results with historical controls.

## 2. Materials and Methods

 The study protocol was approved by the Institutional Review Board of the healthcare facility.

The medical records of patients who underwent surgery for inflammatory frontal sinus disease between the years of 1995 and 2010 were reviewed. There were a total of 3587 frontal sinus operations performed during that period. This included both endoscopic and open approaches to the frontal sinus. The 39 patients (1.1%) among this group who underwent OFSO comprised our study population. Indications for OFSO other than chronic frontal sinusitis and frontal sinus mucocele were excluded; five patients who underwent OFSO for fracture repair or osteoma removal were not included in this study. The surgeries were performed at one institution (*n* = 34; 0.9% of the total group of patients who underwent surgery for inflammatory frontal sinus disease) by seven different surgeons, though all surgeons performed a technique similar to the one described by Montgomery [7–9].

 Data regarding age, gender, date and nature of prior frontal sinus operation(s), preoperative imaging, and preoperative diagnosis were examined. Additionally, type of incision, estimated operative blood loss, and operative complications were recorded. OFSO was recorded as first line therapy only in cases without any prior frontal sinus surgery; salvage OFSO refers to cases with any prior frontal sinus surgery. The outcome of surgery was assessed by symptom resolution through a patient self-filled questionnaire and the need for revision surgery. 

A MEDLINE search was then conducted in a standard fashion to compare the current tabulated results with historical controls.

## 3. Results

 Thirty four patients who underwent OFSO for inflammatory frontal sinus disease were included in this paper; 23 patients underwent OFSO for chronic frontal sinusitis and 11 patients for frontal sinus mucocele (Table 1). There were 18 males and 16 females with an age range of 19 to 76 years (mean age = 49.7 yrs). All patients were imaged preoperatively by CT and 6 patients also had an MRI. Surgery was approached by a bicoronal incision in 19 patients, midforehead incision in 11 patients, and eyebrow incision in 4 patients. Estimated blood loss ranged from 50 mL to 700 mL (mean = 160 mL). There was one immediate operative complication, orbital hematoma that resulted in no long-term sequelae for the patient.

 In patients who underwent OFSO in this study, 56% (19/34) had obliteration as a salvage procedure and 44% underwent OFSO as a first-line surgical therapy (Table 1). The majority of patients with chronic frontal sinusitis (16/23, 70%) underwent OFSO as a salvage surgery after previous frontal sinus surgery failures, while the remaining 30% (7/23) underwent OFSO as a primary surgical treatment for their disease. The dates of these first-line obliteration surgeries spanned the entire study period (1995 to 2010). The reasons cited by the operating surgeons for recommending OFSO as primary surgical treatment included the presence of intrafrontal cell (3 patients), markedly narrowed frontal recess with prominent beak and small frontal sinus (2 patients), and the presence of frontal sinus disease in far lateral locations which would be difficult to access endoscopically (2 patients). One patient who underwent first-line surgery with OFSO required revision OFSO during the study period (20 months after the first OFSO). 

For the patients in whom OFSO was a salvage procedure for chronic frontal sinusitis, the failed surgeries were most often endoscopic approaches to the frontal sinus (11/16, 69%), with the patients presenting between 7 months and 7 years following their prior procedures (Table 2). Two patients (2/16, 12%) failed a prior Lynch procedure; the first patient had this prior procedure one year before OFSO, and the second patient four years before OFSO. Finally, three patients (3/16, 19%) presented with worsening symptoms with history of previous OFSO outside this study period. One of the patients from this last-mentioned subset had undergone OFSO thirty years prior to the revision OFSO, and also had undergone EMLP seven years prior to the OFSO recorded in this study. One patient with chronic frontal sinusitis who underwent salvage obliteration required revision OFSO during this study period, two months following the first OFSO. This patient had undergone greater than twenty endoscopic surgeries by outside surgeons prior to the first OFSO. 

**Figure 1:**
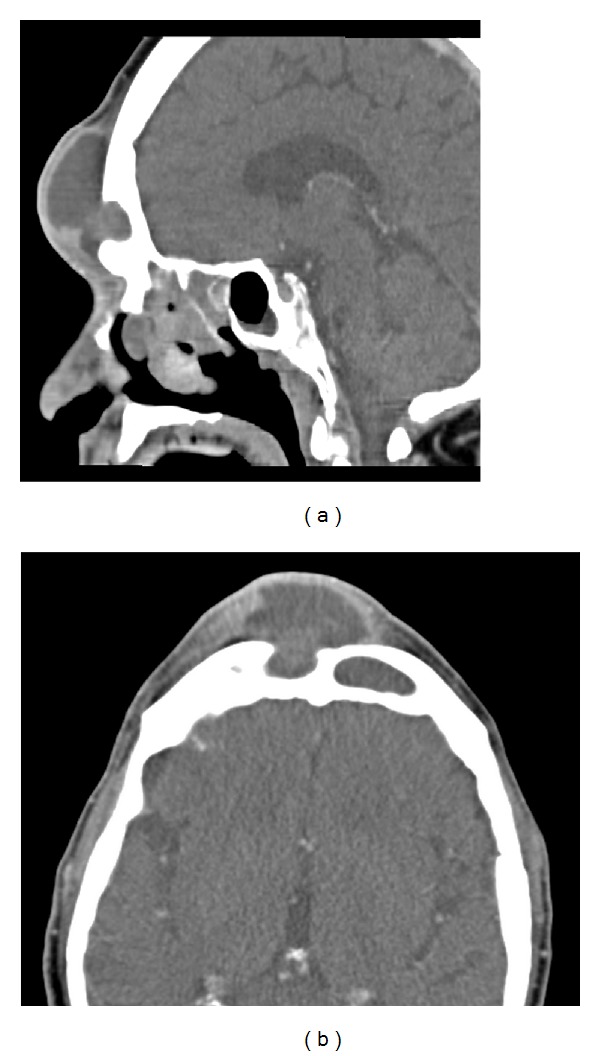
Osteoplastic frontal sinus obliteration (OSFO) in the setting of anterior table erosion. Sagittal (a) and axial (b) CT scan of sixty seven year old man who presented with pansinusitis and right frontal mucopyocele and resulting anterior table erosion. After urgent decompression and six week course of intravenous antibiotics, this patient underwent successful OFSO and has remained symptom-free for eighteen months.

For patients with frontal sinus mucocele, 72% (8/11) had OFSO as a first-line surgical treatment. None of these patients required revision surgery during the study period. Two of these eight patients showed evidence of erosion of the anterior table by CT preoperatively, and four had a compartmentalized mucocele involving the lateral recess of the frontal sinus; for the remaining two patients, the surgeon(s) cited “fear of potential recurrence” as the indication for OFSO. An example of the use of OFSO in the setting of anterior table erosion is shown in Figure 1. Twenty-seven percent (3/11) of patients with mucocele underwent OFSO following previous frontal sinus surgeries: two patients had previous OFSO sixteen and twenty-one years before this study, respectively, and one patient had two endoscopic surgeries three and four years earlier. 

Within the total study population, 15% (5/34) of patients presented for OFSO with history of prior obliteration, with a range of 3 to 30 years between representation. The indication for OFSO in this subset was mucocele in only 2 of the 5 patients. One patient who underwent revision OFSO for mucocele sixteen years after the first also underwent an endoscopic surgery one year following the revision for supraorbital ethmoiditis. 

## 4. Discussion

 Osteoplastic frontal sinus obliteration (OFSO) with abdominal fat has been accepted as the definitive technique for complicated frontal sinus disease for many years, dating back to studies by Goodale and Montgomery [9, 10]. In the comprehensive study by Hardy and Montgomery [10] in which 250 OFSO surgeries were performed with a median followup of 8 years, they noted a revision rate of 4%, while 93% of 208 patients reported full resolution of symptoms. Mucocele formation rate was not known as computerized imaging of the obliterated sinus was not yet available [6]. Morbidity from OFSO primarily related to postoperative infection, abdominal wound complications, and troubling aesthetic changes of the frontal bone have been reported between 12 and 18% [6]. 

 In the time since OFSO was first described, advancements in endoscopic technology have allowed development of multiple endoscopic techniques for frontal sinus disease. A graduated approach utilizing sequentially more advanced endoscopic techniques has been proposed [11]. In the presence of frontal sinus pathology, endoscopic frontal sinusotomy is the first and least involved approach [11]. Frontal sinus drillout, or endoscopic modified Lothrop procedure (EMLP), can be utilized for cases of frontal sinusitis that do not respond to conservative surgical intervention, and frequently involves the removal of the frontal sinus floor as well as superior nasal septum and interfrontal septum [5, 11]. 

Success for EMLP has been reported as 80–93%, similar results as seen for OFSO [5, 11]. Hence, EMLP has gained favor not only for first line therapy but as a salvage technique for patients who have failed OFSO [3]. However, long-term followup from these endoscopic techniques has not yet been possible due to their recent introduction into clinical practice. Given the average time to presentation of failure for OFSO is greater than ten years [12], no study utilizing endoscopic techniques can accurately assess long-term results. The longest follow-up period for endoscopic approaches to frontal sinus disease was published recently. In Friedman et al. [13], a large group of patients who had already been retrospectively presented in Friedman et al. [14] was re-studied with a longer follow-up period (72 months as compared to 12 months originally). Although a similar success rate of symptom improvement was seen in the two studies, only 37.5% of original patients were available for endoscopy to directly assess frontal recess patency [13]. Thus, power was limited since a high percentage of patients were lost.

 Clearly, even in the era of image-guided endoscopic surgery, OFSO still plays an important role in refractory disease. In a more recent study of 43 OFSO procedures, 97% of patients saw resolution of symptoms [15]. This study suggests additional benefit from proper treatment of frontal sinus disease, as 63% of patients also had improvement or resolution of disease in other paranasal sinuses [15]. In a quality-of-life study of patients who underwent OFSO, two thirds of patients (*n* = 39) were satisfied with the surgery, and 70% reported improvement in their presenting symptoms [16]. While maintaining that OFSO is a last resort, Anand et al. [17] also propose a low threshold for performing obliteration depending on the patient's anatomy and extent of disease. This study is comparable to multiple others in which surgical success rates for OFSO is greater than 90%, rates similar to the ones reported originally by Montgomery [9, 10].

Our data suggest that OFSO may still be considered in carefully selected number of cases as an alternative front-line therapy to endoscopic approaches for severe frontal sinus disease in patients with endoscopically inaccessible anatomy. As detailed in Section 2, patients who underwent OFSO constituted a tiny minority (0.9%) compared to those who underwent endoscopic surgery for their frontal sinus disease. In planning the appropriate surgical approach, the anatomy of the frontal recess was carefully analyzed utilizing sagittal CT images in addition to axial and coronal ones, as sagittal cuts improve visualization of the anterior-posterior dimension of the frontal recess, and its relationship to Agger Nasi cells. In this moderately sized study population (*n* = 34), 44% of patients underwent OFSO as their first surgery for inflammatory frontal sinus disease. While more common in the group presenting with frontal sinus mucocele, 30% of the patients described here with chronic frontal sinusitis who failed medical therapy underwent OFSO rather than endoscopic frontal sinus surgery. As was detailed in Section 3, anatomy of the frontal recess, the presence of intrafrontal cells, the size of the frontal sinus, and the presence of frontal sinus disease in far lateral locations which can be difficult to access endoscopically were the main determinants for choosing OFSO as a primary surgical modality. These patients did well postoperatively without the need for additional surgery during the study period, except for one patient who underwent revision obliteration 20 months after the first.

When we analyzed those patients who had first-line OFSO for the treatment of mucoceles, there were multiple factors associated with their disease including the presence of frontal sinus disease in far lateral locations which would be difficult to access endoscopically, the presence of anterior frontal sinus wall erosion, compartmentalized mucoceles, as well as the individual surgeon's experience with this procedure influenced the technique chosen. We had first hypothesized that the distribution of first line OFSO cases would be weighted to the earlier years in the study, since endoscopic techniques became more commonplace during this period. However, the first line cases were evenly distributed throughout the 15-year period, consistent with the conclusion that the nature of disease and surgeon's experience determined the technique. 

In the patient population presented here, as defined by further need for surgical intervention, OFSO allowed a success rate of 91% as only three of 34 patients underwent additional paranasal sinus surgery during the study period (15 years). Two of these patients underwent revision OFSO, and the third additional endoscopic surgery. There was only one complication, orbital hematoma, which was effectively diagnosed and treated, and did not result in any long-term sequelae. 

The data presented contain obvious limitations. The descriptive nature, the presence of multiple surgeons, and varied follow-up times complicate our ability to make broad generalizations. However, the following conclusions can be drawn: OFSO continues to be a safe and effective procedure for treatment of carefully selected patients who suffer from chronic inflammatory frontal sinus disease, and should be included in the preoperative counseling of these patients. The treating surgeon needs to make the appropriate recommendation and decision based on his/her interpretation of the long-term success rate of endoscopic approaches to the frontal sinus including EMLP, the nature and the location of the frontal sinus disease, the specific patient situation (co-morbidities, easy access, and reliability of long-term follow-up), and his/her own surgical experience and skill. In the era of image-guided endoscopic sinus surgery, OFSO is still an important component of a graduated approach to the frontal sinus, and a useful tool both in salvage and first-line surgery for chronic frontal sinusitis and frontal sinus mucocele, specifically a disease that involves the far lateral recesses of the frontal sinus which would be difficult to access endoscopically, the presence of anterior frontal sinus wall erosion, and the individual surgeon's experience. However, as clearly demonstrated in our data, the vast majority of patients with refractory inflammatory frontal sinus disease can be successfully treated with endoscopic surgical approaches. 

## 5. Conclusion

 OFSO remains a valuable surgical technique in the treatment of inflammatory sinus disease. OFSO should be included in the preoperative counseling of patients with frontal sinus disease. The decision between an endoscopic approach and the OFSO technique depends on the surgeon's experience, the overall clinical picture and nature of the disease, and the anatomy of the frontal recess.

## Figures and Tables

**Table 1:** The use of osteoplastic frontal sinus obliteration (OFSO) as first line or salvage therapy correlates with diagnosis.

Diagnosis	Number OFSO patients	First line OFSO	Salvage OFSO
Chronic frontal sinusitis	23	7/23 (30%)	16/23 (70%)
Frontal sinus mucocele	11	8/11 (72%)	3/11 (28%)
Total inflammatory frontal sinus disease patients	**34**	**15/34 (44%)**	**19/34 (56%)**

**Table 2:** The majority of salvage osteoplastic frontal sinus obliteration (OFSO) patients failed prior endoscopic frontal sinus surgery.

Diagnosis	Salvage OFSO	Hx of prior OFSO	Hx of prior ESS	Hx of prior lynch
Chronic frontal sinusitis	16	3/16 (19%)	11/16 (69%)	2/16 (12%)
Frontal sinus mucocele	3	2/3 (67%)	1/3 (33%)	0/3 (0%)
Combined inflamm frontal sinus disease	**19**	**5/19 (26%)**	**12/19 (63%)**	**2/19 (11%)**

Hx: History; ESS: endoscopic sinus surgery; inflamm: inflammatory.
